# Pedunculated focal nodular hyperplasia: a case report, case series, and in-depth surgical, radiological, and histological analysis of a rare phenomenon

**DOI:** 10.1186/s13000-025-01661-y

**Published:** 2025-05-30

**Authors:** Taylor Strange, Joseph M. Gosnell, Peeyush Bhargava, Abdulrahman Al Harbi, Luca Cicalese, Heather L. Stevenson

**Affiliations:** 1 Department of Pathology, University of Texas Medical Branch, 301 University Blvd, Galveston, TX 77555 USA; 2 Department of Radiology, University of Texas Medical Branch, Galveston, USA; 3 Department of Surgery, University of Texas Medical Branch, Galveston, USA

**Keywords:** Pedunculated focal nodular hyperplasia, Liver, Diffusion-weighted imaging, Glutamine synthase

## Abstract

**Background:**

Focal nodular hyperplasia (FNH) is a benign hepatic lesion that rarely presents as an exophytic mass attached by a fibrous stalk (termed pedunculated FNH). This variation poses a challenge to clinicians, with atypical symptoms and imaging.

**Case presentation:**

We describe a 33-year-old female who underwent excision of a pedunculated FNH. On gross examination, the lesion was lobular and vascular with homogenous tan-brown surfaces. Histological examination showed loss of normal liver architecture, abnormal intervening fibrous tracts, dysplastic arteries, and focal steatosis. Immunohistochemical staining with glutamine synthetase resulted in a branching, or “map-like” pattern. These findings were consistent with focal nodular hyperplasia. One of the most sensitive imaging techniques for diagnosing this lesion involves magnetic resonance imaging (MRI) with contrast, which discloses a homogenous mass that is hyperintense during the arterial phase with gradual decrease in intensity during the venous and equilibrium phases. The central stellate scar will often remain hyperintense for a prolonged period of time. On histology, normal hepatic architecture is lost to abnormal fibrotic bands and a characteristic stellate scar. Immunohistochemistry with glutamine synthetase uniquely highlights a map-like pattern that is not seen in other liver lesions.

**Conclusions:**

Due to its atypical presentation and increased risk of complications compared to its intrahepatic counterpart, pedunculated FNH brings unique challenges for diagnosis and therapy. Proper identification of pedunculated FNH is critical for appropriate treatment. Our case highlights the importance of radiological and histopathological studies to accurately identify this lesion, as well as the benefits of surgical removal to prevent serious complications.

## Introduction

Focal nodular hyperplasia (FNH) is the second most common benign hepatic lesion after hemangioma. FNH predominantly occurs in women of childbearing age, and is believed to be a result of abnormal vascular proliferation [1]. This hypothesis is supported by its concomitant appearance with other vascular disorders. While FNH is generally asymptomatic and does not require surgical intervention, it is important to differentiate from other lesions, including hepatic adenoma, which generally does require surgical intervention to avoid complications such as tumor hemorrhage.

Rarely, FNH presents as an exophytic mass, and is termed pedunculated FNH. This variation is characterized by an extrahepatic, well-circumscribed lesion consistent with focal nodular hyperplasia that is attached to the liver by a thin fibrous stalk. This presentation poses a challenge to clinicians as symptoms can range from minimal to severe, with some patients experiencing tumor infarction, bleeding, and compression of surrounding structures. Here, we describe a case of a 33-year-old female with no significant past medical history who presented with abdominal pain, and was found to have a pedunculated FNH. We also provide a review of the current literature of this rare presentation of FNH, as well as useful methods for its identification from a radiographical and histological perspective.

## Materials and methods

**Fig. 1:**
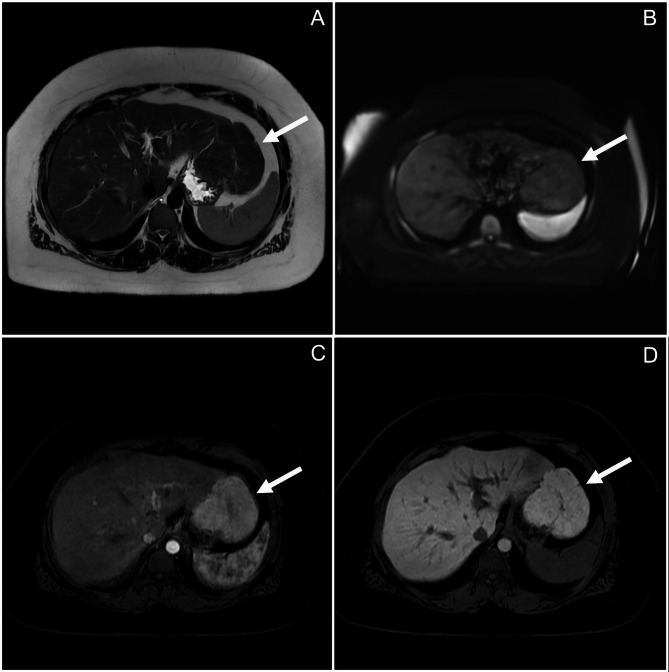
MRI imaging indicated pedunculated focal nodular hyperplasia. The lesion is isointense compared to surrounding hepatic tissue on T2-weighted MRI (**A**) and Diffusion-weighted imaging (DWI, **B**). With injection of Eovist contrast, the lesion became enhanced during the arterial phase (**C**), and remained enhanced after 20 min (**D**), findings that are characteristic for FNH.

A 33-year-old female with a past medical history significant only for a recent Nexplanon injection presented to the ED with abdominal pain. Imaging showed an unusual fullness in the left lateral liver segment, and follow-up imaging at an outside hospital noted several small, benign appearing lesions in both lobes of the liver, along with a large exophytic lesion stemming from the left lobe; all were suspected to be hepatic adenomas. She began following with hepatology who ordered an MRI, which indicated two predominant lesions: one 3.5 cm lesion within segment 4A of the liver, and exophytic lesion measuring 9.0 cm. (Fig. 1) Both lesions were consistent with focal nodular hyperplasia. Liver function enzymes and bilirubin were within normal limits.

After discussion with the patient, it was decided that she would remain on surveillance. However, ten months later, she continued to experience abdominal pain, so excision was scheduled.

During the operation, the 3.5 cm lesion was observed under the falciform ligament in segment 4, and was treated intraoperatively with microwave ablation. The exophytic lesion was identified and found to have several thick veins surrounding it, complicated by intraoperative bleeding, which was controlled with numerous silk ties. The lesion was excised entirely and submitted to pathology.

## Results

**Fig. 2:**
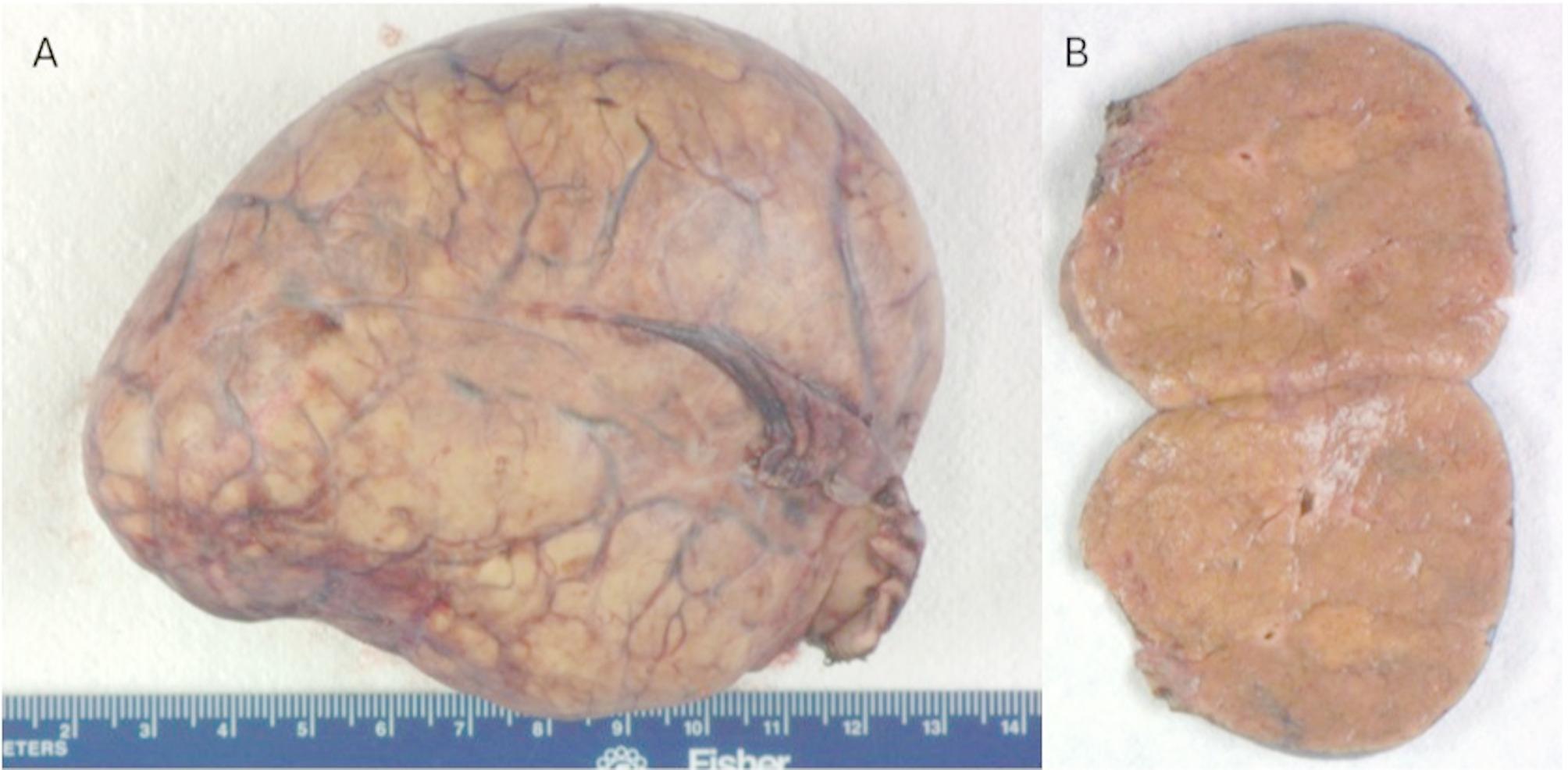
Gross examination revealed an encapsuled lesion with a highly vascular surface (**A**). Serial sectioning (**B**) showed homogenous tan-brown surfaces.

**Fig. 3:**
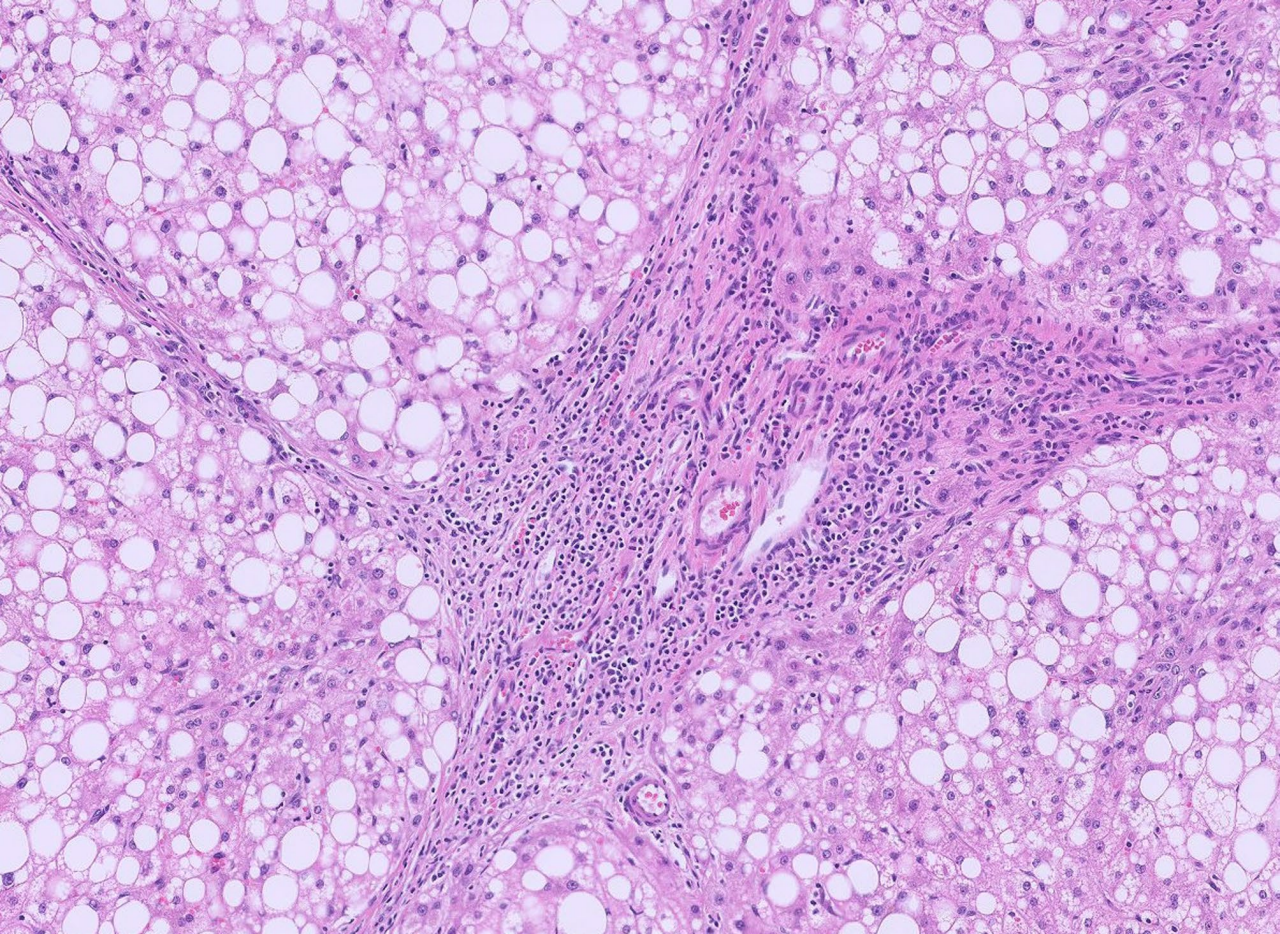
Histology displayed extreme macrovesicular steatosis, intervening abnormal fibrous tracts, and dysplastic arteries.

**Fig. 4:**
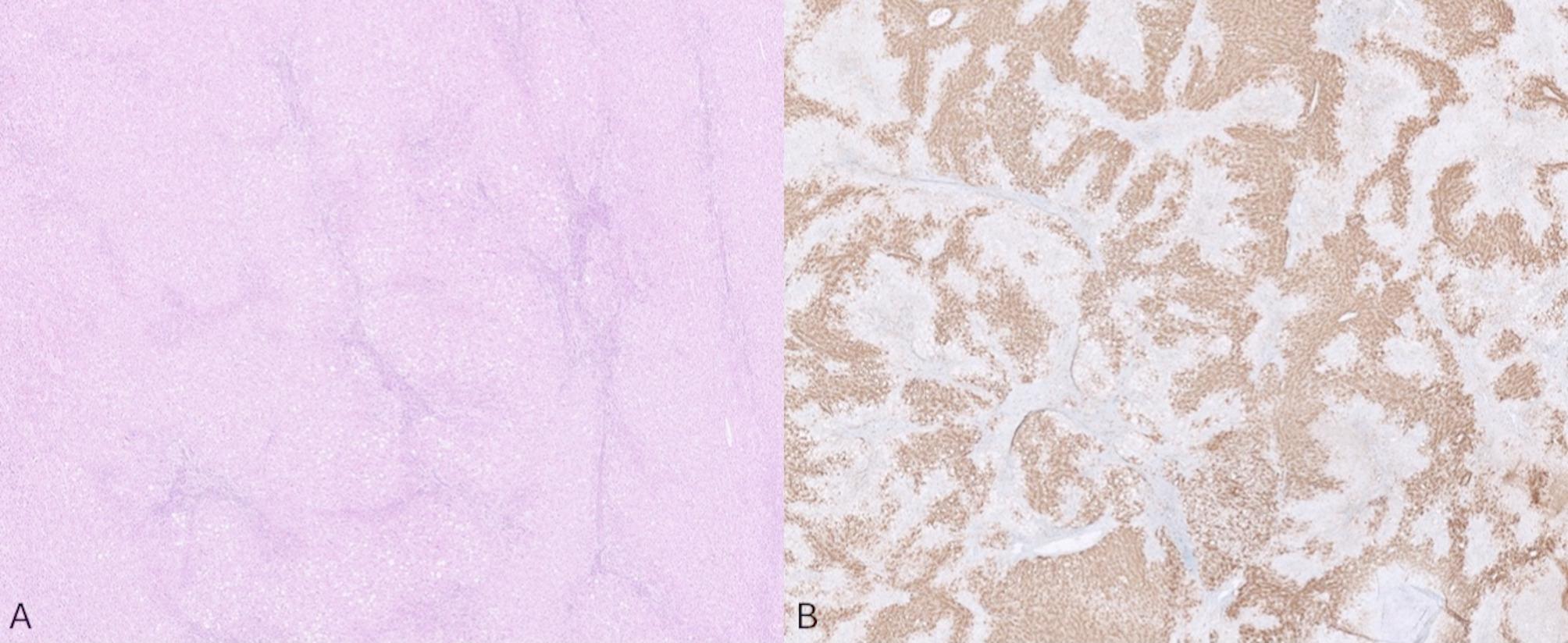
Loss of normal hepatic architecture with bands of atypical fibrous tracts within the tumor (**A**). Staining with glutamine synthetase resulted in a characteristic “map-like” pattern commonly observed in focal nodular hyperplasia (**B**).

On gross examination, the lesion was lobular and vascular. Serially sectioning the specimen revealed homogenous tan-brown surfaces. (Fig. 2) Histological examination showed loss of normal liver architecture, abnormal intervening fibrous tracts, dysplastic arteries, and focal steatosis. Immunohistochemical staining with glutamine synthetase resulted in a branching, or “map-like” pattern. (Figs. 3 and 4) The patient was ultimately diagnosed with both intrahepatic FNH and pedunculated FNH. She was discharged three days later with no post-operative complications.

## Discussion

Focal nodular hyperplasia (FNH) is a benign nodular lesion of the liver that most commonly presents in middle-aged women. It is best characterized by its central stellate scar, which is composed of dense collagen surrounding a fibrovascular core, and is appreciated both via histology and imaging. It often displays medium-sized vessels that overtake the normal hepatic architecture [1].

The exact pathophysiology of FNH is not known. It has been theorized that female sex hormones play a role to some degree, particularly since these lesions have a predilection for women of childbearing age. Oral contraceptives have been hypothesized to contribute to FNH growth and size, but this remains an ongoing source of debate. Another potential etiology is abnormal vascular proliferation, fortified by the documented associations between FNH and vascular disorders (e.g., Osler-Weber-Rendu disease, Budd-Chiari syndrome, and congenital abnormalities of the ductus venosus ) [2–4] as well as mutations in genes such as *ANGPT1* and *ANGPT2* [1, 5]. According to this theory, FNH is a consequence of hepatocytes generating nodules around an abnormal cumulation of vessels. Such lesions arising in the context of other vascular disorders have been termed FNH-like lesions [6]. 

Other molecular associations have been studied, although the majority of FNH lesions demonstrate remarkable genetic stability. For example, upregulation of genes related to collagen production and cell adhesion (likely contributing to the increased fibrosis found in these lesions) has been identified [6]. In contrast to other hepatic tumors, FNH does not commonly express beta-catenin (CTNNB1) or HNF1α mutations, which are seen in hepatocellular carcinomas and adenomas, respectively [5, 7]. Ironically, however, FNH lesions sometimes display activation of the beta-catenin pathway, such as via increased presence of unphosphorylated beta-catenin and positive identification of mRNA sequences known to be regulated by beta-catenin, despite their genetic profile [6]. Additionally, one study found increased expression of the TGF-β1 and PDGFA/PDGFRB in focal nodular hyperplasia lesions when compared to benign liver tissue, particularly adjacent to the central scar. These findings suggest upregulation of the TGF-beta pathway [6, 8].

Seldomly, rather than developing within the liver, FNH lesions develop within the abdominal cavity, remaining associated to the liver by a mere thin fibrous stalk. This variation is termed pedunculated focal nodular hyperplasia. Due to its rare presentation, few case reports of this entity have been published (Table 1) [9–20]. Twelve total published cases were available online. All patients in these cases were female, and apart from two children (three and nine years old), all patients were middle-aged (ranging from 20s to 40s). The most common presentations were either asymptomatic (e.g., lesions of pedunculated FNH found incidentally while acquiring imaging for an unrelated comorbidity) or abdominal pain. Lesions ranged from 2.9 to 16.0 cm in greatest diameter, with tumor stalks showing no apparent predilection for any hepatic region. All lesions were removed surgically, and were confirmed as focal nodular hyperplasia via histopathological and immunohistochemical evaluation. Of these cases, only two were noted to involve intraoperative complications (cholecystectomy due to shared vasculature between gallbladder and FNH lesion, stalk torsion), and none had documented complications following discharge.

**Table 1:** Previous case reports of pedunculated focal nodular hyperplasia

Authors	Patient Demographics	Presentation	Origin	Tumor size	Complications
Brouquet et al. (1985) [9]	40-year-old female	Found incidentally during hysterectomy	Segment 4	N/a	None
Bader et al. (2001) [19]	Middle-aged female	Found incidentally via ultrasound	Segment 5	4.5 cm	N/a
Wasif et al. (2008) [10]	48-year-old female	Right upper quadrant pain	Segment 4b	3.2 cm	None
Badea et al. (2015) [20]	29-year-old female	Abdominal pain	Hilum	8 cm	Shared vascularity with gallbladder requiring cholecystectomy
Reddy et al. (2015) [11]	35-year-old female	Abdominal pain	Segment 3	2.9 cm	None
Zeina and Glick (2016) [12]	25-year-old female	Abdominal pain and nausea	Segment 2	4.8 cm	N/a
Koowal et al. (2018) [13]	3-year-old female	Painless palpable mass on physical exam	Right hepatic lobe	5.9 cm	N/a
Navarini et al. (2020) [14]	26-year-old female	Abdominal pain	Left hepatic lobe	13 cm	Pedicle torsion causing tumor ischemia
Ben Ismail et al. (2021) [15]	38-year-old female	Found incidentally during epigastric hernia repair	Segment 3	3 cm	None
Akaguma et al. (2023) [16]	35-year-old female	Found incidentally during prenatal ultrasound	Segment 6	7 cm	None
Nandy et al. (2023) [17]	40-year-old female	Abdominal pain	Segment 5	16 cm	None
Moayerifaret al. (2024) [18]	9-year-old female	Abdominal pain and palpable mass	Segment 6	9.2 cm	None

Pedunculated FNH is believed to have a similar etiology to intrahepatic FNH. However, due to its rare presentation and its existence external from the liver, it poses unique challenges for the surgeon, radiologist, and pathologist. Both the radiologist and pathologist must confirm this exophytic lesion is indeed of hepatic origin, and that the lesion is benign in nature. The surgeon must determine if removal of the lesion is necessary and must avoid damage to surrounding organs. Several key considerations can aid in an accurate diagnosis:

### Clinical presentation

When small, pedunculated FNH may be asymptomatic like its intrahepatic counterpart. However, large exophytic variants of FNH may encroach on surrounding structures, resulting in abdominal pain, nausea, and a tender, mobile abdominal mass on physical exam. Additionally, the lesion may undergo bleeding, torsion, infarction, or rupture, any of which could result in a surgical emergency [21]. 

### Radiography

Imaging plays a critical role in determining the identity and extent of pedunculated FNH. Notably, the radiologist must be confident the lesion is not an exophytic variant of other hepatic lesions, such as hepatic adenomas, hepatic cysts, hepatocellular carcinomas, or metastatic lesions. Ultrasound, computed tomography (CT), and magnetic resonance imaging (MRI) are all imaging modalities that can aid in identifying both intrahepatic and pedunculated FNH [2]. Of the three, MRI with contrast is most specific and sensitive [2]. When contrast is given with an MRI, classic findings of FNH include a homogeneous lesion with intensity that varies with the phase and whether T1 or T2 weighted images are utilized [22]. FNH classically appears as isointense to slightly hypointense on T1 weighted images, and isointense to slightly hyperintense on T2 weighted image. It is usually hyperintense during the arterial phase with a gradual decrease in intensity during the venous and equilibrium phases. It should retain contrast with specific hepatobiliary contrast (e.g. Eovist or Primosvist). Additionally, its characteristic central stellate scar will often remain hyperintense for a prolonged period. In contrast, for example, a hepatic adenoma will also appear homogeneous, but will be hyperintense for both the arterial and venous phase of T2 weighted imaging [2, 19, 23]. (Table 2)

**Table 2:** Classic radiological findings of liver lesion [19, 22, 24]

Lesion	Pre-contrast T1 weighted imaging findings	T2 weighted imaging findings
Focal nodular hyperplasia	Lesion is isointense to hypointense with a hypointense central stellate scar	Lobulated lesion that is hyperintense during arterial phase with gradual decrease in intensity during venous and equilibrium phases. Delayed enhancement of central stellate scar
Hemangioma	Initial hypointensity with potentially gradual nodular enhancement over time	“Light bulb” T2 hyperintensity; peripheral enhancement that fills in during transition from arterial to equilibrium phase
Hepatic adenoma	Homogenous hypointensity compared to surrounding liver parenchyma	Hyperintense throughout arterial and venous phases
Hepatocellular carcinoma	Homogeneous hypointensity compared to surrounding liver parenchyma	Arterial enhancement with gradual loss or “washout” of contrast during venous phase; delayed appearance of hyperintense pseudocapsule

It is crucial that an exophytic FNH lesion be differentiated from other potential abdominal masses originating from a fibrotic stalk, such as tumors of the retroperitoneum, gastrointestinal tract, or adrenal glands [20]. The classic features mentioned previously should still be present; however, diagnosis can still be challenging since the lesion is in an abnormal anatomical location and the pedicle is not always visible on imaging. Thus, surgical intervention and histological evaluation often plays the final diagnostic role.

### Pathology

Grossly, as with classic intrahepatic FNH, pedunculated FNH will present as a well-circumscribed, though nonencapsulated, solitary lesion. It is often described as tan-yellow and lobulated in appearance [21]. On histology, normal hepatic architecture is lost to the fibrotic bands formed by the lesion, and the characteristic stellate scarring should be apparent (thus biopsies with small volumes of tissue can be challenging if the scar is not sampled) [25]. Cirrhosis is absent. While normal portal tracts will be absent, there may be bile ductular proliferation and arteries with intimal fibrosis [25]. Other potential histological findings include ballooning or Mallory Denke bodies in the surrounding viable hepatocytes. As in our case, immunohistochemistry is also useful. In particular, glutamine synthetase uniquely highlights a map-like pattern on only FNH specimens. In contrast, lesions such as hepatocellular adenoma will stain diffusely with glutamine synthetase, thus lacking the characteristic map-like pattern (Table 3) [26, 27]. 

**Table 3:** Comparison of immunohistochemistry staining patterns among the most common hepatic lesions [27–30].

	Glutamine synthetase	CK7	CK19	Glypican-3
Benign liver	Perivenular pattern	Highlights cholangiocytes	Highlights cholangiocytes	Negative or weak
Focal nodular hyperplasia	Map-like pattern	Positive in ductular reaction	Positive in ductular reaction	Negative or weak
Hemangioma	Negative or weak	Negative	Negative	Negative or weak
Hepatic adenoma	Diffuse	Negative or weak	Negative or weak	Negative or weak
Hepatocellular carcinoma	Diffuse	Positive in ductular reaction	Positive in ductular proliferation	Positive

## Conclusion

Focal nodular hyperplasia is a benign hepatic lesion that often is only found incidentally and generally follows an indolent course. Its pedunculated variant, on the other hand, is a much rarer phenomenon that brings unique challenges for diagnosis and therapy. Our case highlights the importance of radiological and histopathological studies to accurately identify this lesion, as well as the benefits of surgical removal to prevent serious complications.

## Data Availability

No datasets were generated or analysed during the current study.

## Abbreviations

FNH: Focal nodular hyperplasia
MRI: Magnetic resonance imaging
CT: Computed tomography
ED: Emergency department
